# Cdc42 Effector Protein 2 (XCEP2) is required for normal gastrulation and contributes to cellular adhesion in *Xenopus laevis*

**DOI:** 10.1186/1471-213X-4-13

**Published:** 2004-10-08

**Authors:** Karen K Nelson, Richard W Nelson

**Affiliations:** 1 Biology Department, Wabash College, 301 W. Wabash Ave., Crawfordsville, IN 47933, USA

## Abstract

**Background:**

Rho GTPases and their downstream effector proteins regulate a diverse array of cellular processes during embryonic development, including reorganization of cytoskeletal architecture, cell adhesion, and transcription. Changes in the activation state of Rho GTPases are converted into changes in cellular behavior by a diversity of effector proteins, which are activated in response to changes in the GTP binding state of Rho GTPases. In this study we characterize the expression and function of one such effector, XCEP2, that is present during gastrulation stages in *Xenopus laevis*.

**Results:**

In a search for genes whose expression is regulated during early stages of embryonic development in *Xenopus laevis*, a gene encoding a Rho GTPase effector protein (Xenopus Cdc42 effector protein 2, or XCEP2) was isolated, and found to be highly homologous, but not identical, to a *Xenopus *sequence previously submitted to the Genbank database. These two gene sequences are likely pseudoalleles. XCEP2 mRNA is expressed at constant levels until mid- to late- gastrula stages, and then strongly down-regulated at late gastrula/early neurula stages. Injection of antisense morpholino oligonucleotides directed at one or both pseudoalleles resulted in a significant delay in blastopore closure and interfered with normal embryonic elongation, suggesting a role for XCEP2 in regulating gastrulation movements. The morpholino antisense effect could be rescued by co-injection with a morpholino-insensitive version of the XCEP2 mRNA. Antisense morpholino oligonucleotides were found to have no effect on mesodermal induction, suggesting that the observed effects were due to changes in the behavior of involuting cells, rather than alterations in their identity. XCEP2 antisense morpholino oligonucleotides were also observed to cause complete disaggregation of cells composing animal cap explants, suggesting a specific role of XCEP2 in maintenance or regulation of cell-cell adhesion in early embryos. This loss of cell adhesion could be rescued by co-injection with a morpholino-insensitive version of the XCEP2 mRNA.

**Conclusions:**

XCEP2 appears to be an essential component in the early developmental program in *Xenopus laevis*. XCEP2 is involved in maintenance of cell-cell adhesion, and as such may constitute a regulatory component that could help to balance the need for tissue integrity and plasticity during the dynamic cellular rearrangements of gastrulation.

## Background

Vertebrate gastrulation depends upon exquisite regulation of diverse morphogenetic processes including changes in cell shape, cellular adhesion and migration. For example, in *Xenopus laevis *coordinated changes in cell shape and motility guide the initial formation of the dorsal lip, the initial site of mesodermal involution [1,2]. Later, the completion of involution of prospective mesodermal cells is strongly influenced by biomechanical forces generated by convergent extension of dorsal mesodermal cells [3]. Convergent extension involves mediolateral elongation of trunk mesoderm cells, followed by medially-converging intercalation. While convergent extension of dorsal mesoderm is a major driving force for the extension and completion of involution movements, epiboly of the non-involuting ectoderm (driven by radial intercalation of cells of the blastocoel roof) is also necessary for efficient gastrulation [4]. The coordinated changes in cell shape and cell migration associated with gastrulation are influenced by diverse processes, including large-scale remodeling of cytoskeletal architecture and precise modulation of cell-cell and cell-matrix associations.

The molecular basis for initiating and regulating the complex cellular movements of vertebrate gastrulation is only beginning to be discerned. Recent work has shown Wnt proteins as key upstream regulators in gastrulation movements. In *Xenopus laevis *and zebrafish, non-canonical Wnt signaling on the dorsal side of developing embryos directly initiates the cellular rearrangements and migration that contribute to convergent extension of involuting mesoderm [5,6]. Other Wnt-mediated signals activate a distinct protein kinase C-dependent signaling pathway, which appears to affect Cdc42 signaling and to provide cues that maintain tissue separation during gastrulation [7,8]. Inactivation of this PKC-dependent pathway causes ineffective separation of involuting mesoderm from overlying ectoderm in *Xenopus*, resulting in severe gastrulation defects [8]. As with the planar cell polarity (PCP) pathway in *Drosophila *[9], vertebrate Wnt signals during gastrulation can involve signaling through the strabismus and JNK (JUN N-terminal Kinase) proteins [10,11], and affect cellular morphology and migratory behaviors through the activities of a diversity of Rho family GTPases [10,12-14].

Experiments perturbing the activity of Rho GTPases have suggested important regulatory roles for these proteins during gastrulation in *Xenopus *[7,10,12,14]. Upon activation, Rho GTPases are known to interact with a variety of downstream effector proteins which in turn mediate changes in actin and microtubule cytoskeletal architecture [15], cell adhesion [16], cell migratory behaviours [17], vesicular transport [18] and signalling [19-21]. Regulation of the coordinated cellular and tissue level processes involved in gastrulation appears to involve multiple Rho GTPases (including Rho, Rac and Cdc42) and multiple downstream effector proteins. However, we are only currently beginning to discern the identities and functions of the repertoire of effector proteins that specifically relay Rho GTPase signals involved in the initiation and coordination of gastrulation movements.

Regulation of cell-cell adhesion is thought to be of particular importance during gastrulation movements in *Xenopus*, when the integrity of the elongating mesodermal sheet must be maintained at the same time that major cellular rearrangements are occuring. Downregulation of C-cadherin-mediated cellular adhesion has been observed to correlate with convergent extension movements of mesodermal cells [22]. This change in C-cadherin mediated adhesion between blastomeres occurs with no detectable change in cell surface expression of the C-cadherin protein. While the precise mechanism of regulation in this developmental context remains unclear, it is known that lateral clustering of cadherins (mediated through interactions between cytoplasmic tails of cadherin proteins) is capable of strengthening adhesive function [23]. In addition, it is also known that Rho GTPases can act through the IQGAP protein to regulate cadherin-mediated adhesion [24-27]. Recent chararacterization of the developmental pattern of IQGAP expression in *Xenopus *embryos suggests that analogous upstream regulatory mechanisms may operate during *Xenopus *gastrulation [28].

In this study, the embryonic expression pattern and function of a *Xenopus *gene encoding a member of the recently characterized CEP/BORG family of RhoGTPase effector proteins [29,30] is explored. These proteins have been shown to have distinct effects on cytoskeletal architecture, cellular morphology, adhesion and migratory behaviours [29,30], and have also been shown to regulate septin function during cytokinesis [31,32].

*Xenopus *Cdc42 Effector Protein 2, hereafter referred to as XCEP2, is shown here to be developmentally regulated during early embryonic stages, with diffuse expression of mRNA in the animal region that diminishes during late gastrula to neurula stages. Experiments employing morpholino-mediated inhibition of translation suggest that expression of this protein is essential for normal gastrulation movements, and that its activity is required for maintenance of cell-cell adhesion between blastomeres of animal cap explants.

## Results

### *Xenopus laevis *cdc42 effector protein 2 (XCEP2) is present in two allelic forms, and its expression is developmentally regulated during early embryogenesis

**Figure 1:**
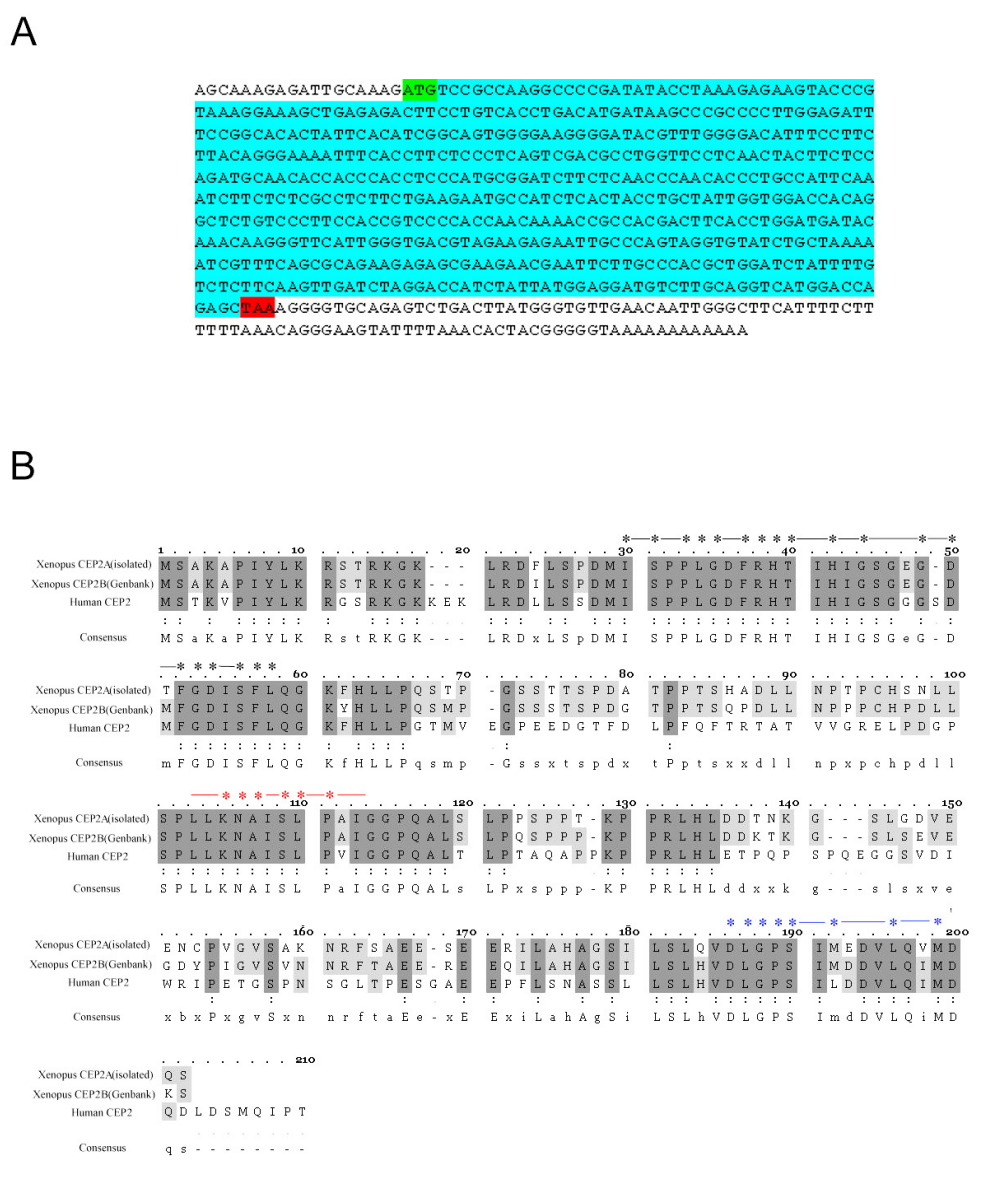
**XCEP2 Nucleic Acid and Amino Acid Sequences. **(A) The nucleic acid sequence of the XCEP2A cDNA is shown. Sequences encoding the start methionine (green), the stop codon (red) are indicated. The protein coding region is shown in blue. (B) Amino acid sequences of the two *Xenopus *pseudoalleles of CEP2, along with the mammalian (human) sequence, are shown. Asterisks indicate highly conserved residues within the CRIB domain (black asterisks, lines), the CI domain (red asterisks, lines) and the CII domain (blue asterisks, lines) in the CEP-2 protein. Dark gray shading indicate residues conserved in all three species; light gray areas indicate residues that are conserved between two of the three species listed. Vertically oriented pairs of dots (:) indicate positions where amino acid identity is conserved in all three sequences.

A differential display approach was employed to reveal genes that are developmentally regulated during the transition from blastula to neurula. This screen allowed for the isolation of numerous cDNA fragments corresponding to differentially regulated mRNAs present in the *Xenopus *embryo. The sequence of one of these fragments was found to be highly homologous to the translational start site region of database cDNA sequences encoding Cdc42 effector protein 2. A full length cDNA corresponding to our isolated fragment was amplified using 3' RACE PCR, cloned and sequenced. An alignment of the predicted amino acid sequence for our isolated full length cDNA sequence with human [29] and *Xenopus *(accession number BC045241) CEP2/BORG1 genes from Genbank revealed significant homology (see Figure 1), particularly within and immediately surrounding the conserved CRIB, CI and CII domains [29,30,33]. When comparing the two *Xenopus *sequences, there were a significant number of positions where the amino acid sequence derived from our isolated cDNA sequence differed from that found in the Genbank sequence (15% of the amino acids were non-identical). These differences were found to occur almost exclusively in regions of the protein outside the three conserved domains (CRIB, CI and CII) that define the CEP family of effector proteins. Given the allotetraploid genetic ancestry of *Xenopus laevis*, and the degree of similarity between the sequences, it is likely that these two sequences constitute pseudoalleles of the same gene. We choose to refer to our isolated pseudoallele as XCEP2A and the database allele as XCEP2B.

**Figure 2:**
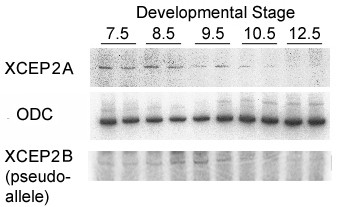
**Temporal regulation of XCEP2 during pre-neurula stages of embryonic development. **Semi-quantitative RT-PCR was conducted on equivalent amounts of RNA derived from the indicated developmental stages. XCEP2A was amplified using XCEP2A forward and reverse primers (see **Methods**). Ornithine Decarboxylase (ODC) serves as a loading control (middle panel). RT-PCR amplification to reveal expression of the alternative pseudoallele (XCEP2B), using XCEP2B forward and reverse primers, is shown in the lower panel.

**Figure 3:**
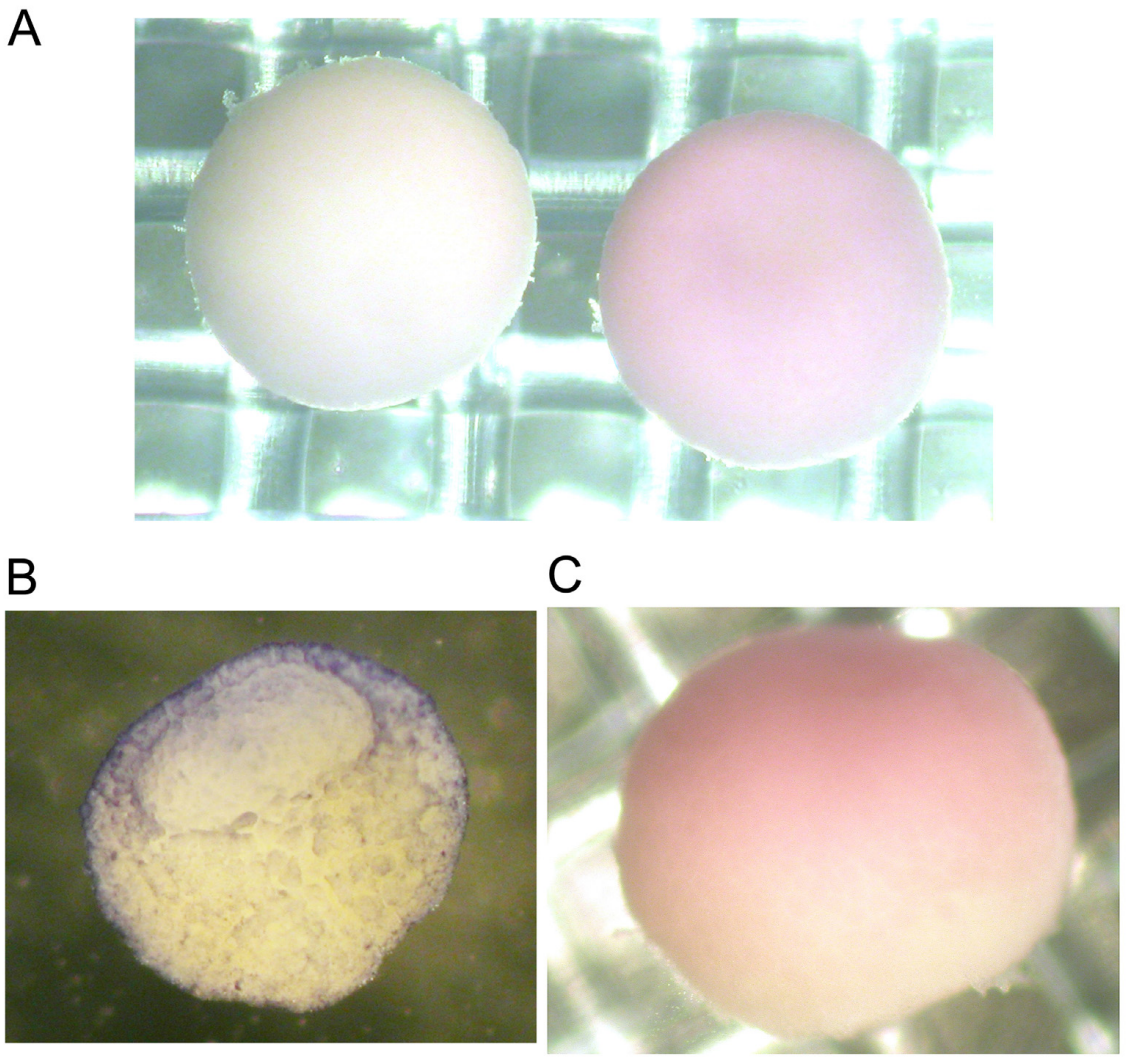
**Spatial Localization of XCEP2 mRNA. **(A) Animal pole view *in situ *hybridization of embryos fixed at late blastula stage with digoxygenin-labeled antisense XCEP2A antisense probe exhibit diffuse staining across the animal region of the embryo (right). An XCEP2A sense probe served as a negative control, and showed minimal background staining (left). (B) A blastula stage embryo, cut to reveal a cross-section, reveals the animal/vegetal gradient of antisense XCEP2 probe. The blastocoel cavity is visible in the upper portion of this embryo. (C) A side view of an antisense XCEP2A probed embryo, revealing an animal (top) to vegetal (bottom) gradient of staining.

Semi-quantitative RT-PCR was conducted on staged RNA samples using primers specific to the 5' untranslated sequence of the isolated XCEP2A cDNA. This analysis revealed the presence of maternally-derived XCEP2A mRNA prior to the onset of zygotic transcription (embryonic stage 7.5), maintenance of this expression level through mid-gastrula stages, with a downregulation of mRNA expression starting at stage 10.5 and becoming more marked by stage 12.5 (see Figure 2). RT-PCR analysis of the expression of the other pseudoallele (XCEP2B) revealed an expression pattern similar to XCEP2A (see Figure 2, lower panel), with an almost complete loss of detectable expression by stage 12.5. Both patterns correspond to that observed for the amplified fragments originally isolated from the original differential display gels (data not shown). *In situ *hybridization revealed that XCEP2A mRNA (corresponding to our isolated pseudoallele) is distributed uniformly over the animal hemisphere of the embryo at blastula and early gastrula stages (see Figure 3A and 3B), with an animal to vegetal gradient in staining evident (Figures 3B and 3C). Over time, there is a diminution of the intensity but no change in the general expression pattern as the embryo transitions from blastula to gastrula stages (data not shown).

### Morpholino oligonucleotides directed against XCEP2 cause gastrulation defects

**Figure 4:**
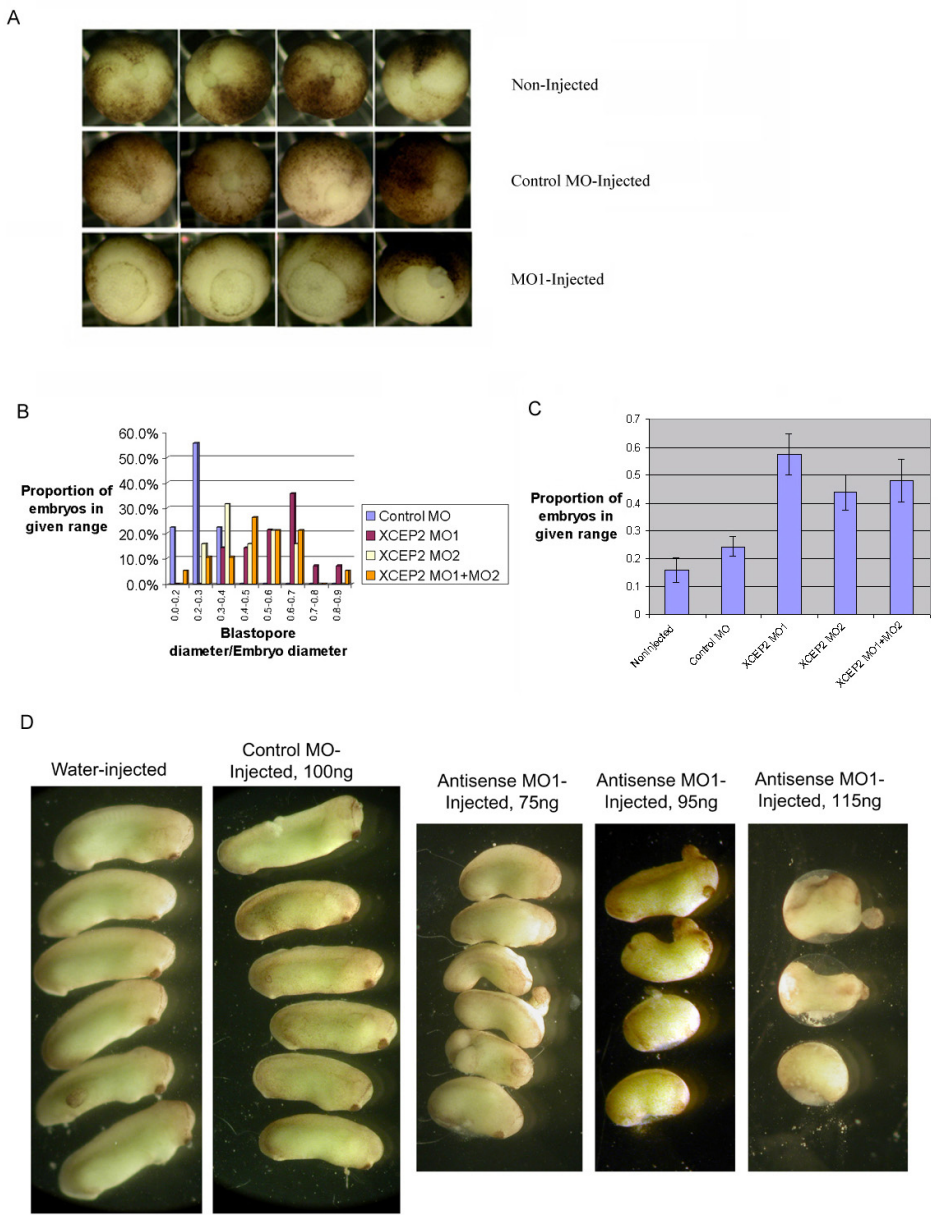
**Effects of antisense XCEP2 morpholino oligonucleotide on gastrulation. **(A) Injected embryos received 100 ng of morpholino, delivered to the animal region of 1-cell embryos. Typical gastrula stage embryos for each of three experimental conditions (non-injected, control morpholino and MO1 antisense injected) are shown. (B) The distribution of blastopore-to-embryo diameter ratios for each experimental condition. 18–20 embryos were assessed for each condition. In the combined "MO1+MO2" condition, 50 ng of each morpholino were co-injected. (C) Average blastopore to embryo diameter ratios for each of the control and experimental conditions, +/- 1SEM, are shown. (D) Effects of antisense morpholinos on post-neurulation embryonic development are shown. Antisense XCEP2-injected embryos exhibit dose-dependent abnormalities suggestive of gastrulation and/or convergence extension defects. Representative viable embryos, at Stage 26–27, from each control and experimental condition are shown. Several embryos in the MO1 treated groups exhibited varying degrees of spina bifida. Embryonic viability for the water, control morpholino, and the three doses of antisense XCEP2 morpholino (75 ng, 95 ng and 115 ng) were 90%, 90%, 81%, 63%, and 26%, respectively (20–30 embryos injected for each condition).

In order to assess the functional role of XCEP2 in early embryogenesis, microinjections were conducted with antisense morpholino oligonucleotides, MO1 and MO2 directed to the translational start site region of one or both of the two XCEP2 pseudoalleles. MO1 is targetted to XCEP2A, and MO2 is targetted to the XCEP2B sequence found in the Genbank database. For this experiment, a total of 100 ng of morpholino oligonucleotide was injected into the animal region of one-cell embryos. At time points just prior to blastopore closure (~embryonic stage 12), MO1 antisense oligonucleotides pseudoalleles elicited significant gastrulation delay as compared to an identical dose of control morpholino oligonucleotide (See Figure 4A). A comparison of the effects of each morpholino, and the combined effects of co-injection of both morpholinos is shown in Figure 4B. The severity of gastrulation delay was quantified at late gastrulation by comparing the ratio of blastopore diameter to embryo diameter in antisense-injected, control injected and non-injected embryos. The mean ratio, and the distribution of ratios differed significantly between control injected and antisense morpholino injected groups (See Figure 4B and 4C). MO2 was slightly less effective than MO1 in producing gastrulation delay. Small differences between the effects of the various injection conditions suggest that either the MO2 morpholino is less efficient at translational inhibition, or that the pseudoallele targeted by MO2 may be expressed at comparatively lower levels. The combined MO1+MO2 condition did not produce more severe defects than the individual morpholino injections, perhaps because the dose of each morpholino in the combined condition was half that in either of the single morpholino conditions (all injections were 100 ng, the combined condition included 50 ng of each morpholino). Many of the more severely affected embryos at this dose did not complete blastopore closure, and showed lethal defects that precluded normal neurulation. Embryos that survived treatments of between 75 and 115 ng of antisense morpholino (MO1) were found to have phenotypes consistent with gastrulation defects, including significant shortening of the AP axis and multiple instances of spina bifida (see Figure 4D). The phenotypes we observe share significant similarity to defects caused by specific inhibition of convergent extension in mesoderm [34]. At a higher dose (100 ng injected into each blastomere of 2-cell stage embryos, 200 ng total injection), embryonic lethality was observed consistently in practically all treated embryos prior to neurulation (see Figure 8A, lower panel).

**Figure 5:**
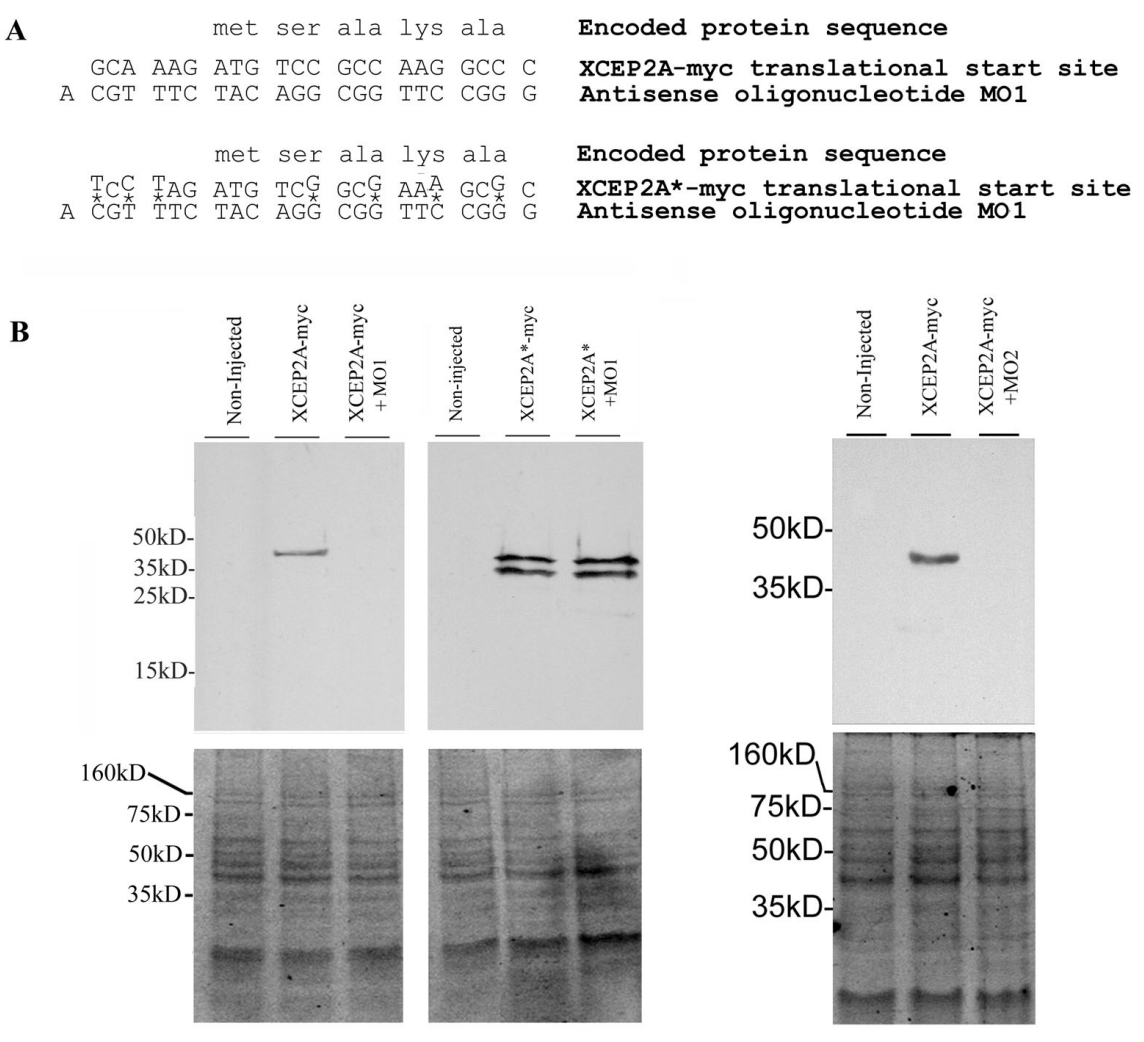
**Antisense XCEP2 morpholino oligonucleotides specifically inhibit translation from XCEP2 mRNA**. (A) Schematic diagram indicating mutations (designated by asterisks) in the morpholino target site of XCEP2*-myc mRNA that would be expected to inhibit strong interactions with the antisense XCEP2 morpholino (MO1). (B) 0.7 ng of mRNA encoding myc-tagged XCEP2 (either the normal XCEP2-myc or the mutated XCEP2*-myc) was injected alone or in combination with 80 ng of antisense XCEP2 morpholino oligonucleotide (MO1) into the animal pole of 1-cell embryos. XCEP2 protein was detected in embryo extracts (prepared at stage 8.5–9) by Western Blotting using anti-myc monoclonal antibody 9E10 (upper panels). Equivalency in protein loads in the Western Blot lanes are indicated by the similarity in staining intensity of protein bands in the corresponding SYPRO Ruby-stained gels in the lower panels.

The antisense morpholino oligonucleotides used in these studies were found to elicit specific translational inhibition of XCEP2. Injection of antisense morpholino against our isolated pseudoallele resulted in largescale inhibition of translation of a myc-tagged fusion protein from co-injected mRNA (see Figure 5B). A modified mRNA (XCEP2A*-myc) encoding a C-terminal myc tagged XCEP2A fusion protein, with seven point mutations in the 5' untranslated region and at the 3^rd ^base of selected codons in the translated region (see Figure 5A), was found to be insensitive to inhibition by antisense morpholino oligonucleotide MO1 (See Figure 5B), demonstrating sequence specificity of the translational inhibition effect. XCEP2A* myc migrates as a doublet, which probably arises due to inefficient translational initiation at the true start codon of the mutated mRNA and some initiation at the second AUG codon, encoding methionine at position 29 (see Figure 1B). While MO1 does not inhibit translation from the multiply mutated XCEP2A* myc mRNA, we did find that MO2, which is designed to be complementary to the database pseudoallele, was capable of significantly reducing translation from mRNA corresponding to our isolated pseudoallele (XCEP2A-myc). This suggests that under these conditions, the two-nucleotide mismatch between MO2 and our XCEP2A-myc mRNA does not preclude translational inhibition (to compare sequences, see **Methods**, "Morpholino Oligonucleotides"). Given this observation, it is possible that injection of either antisense XCEP2 morpholino significantly reduces the endogenous expression of both pseudoalleles. In addition to the dosage issue in the combined condition (noted above), cross-inhibition of this type could contribute to the observed lack of additive effects in the combined MO1 + MO2 condition.

**Figure 6:**
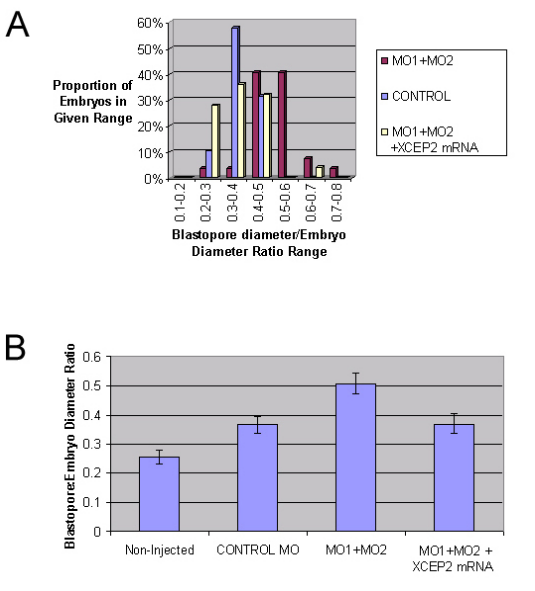
**Rescue of the antisense XCEP2 morpholino phenotype by XCEP2 mRNA co-injection. **Injected embryos received 100 ng of morpholino oligonuceotide in the animal region of both blastomeres in two-cell stage embryos. MO1 and MO2 oligonucleotides were mixed at a molar ratio of 3:1, respectively. 20–30 embryos were assessed for each condition. (A) The distributions of blastopore to embryo diameters for the indicated experimental conditions are shown. The rescuing XCEP2 mRNA (XCEP*-myc) sequence was altered such that antisense morpholino oligonucleotides would be expected to bind inefficiently. (B) Average blastopore to embryo diameter ratios, +/- 2 SEM, are indicated for each experimental condition.

The blastopore closure delay phenotype observed in antisense morpholino injected embryos could be completely rescued by co-injection with 0.7 ng of the XCEP2A*mRNA, which is insensitive to translational inhibition (see Figure 6). This result suggests that the observed effects of antisense morpholino oligonucleotides on gastrulation are not due to inhibition of a non-XCEP2 gene product, or to non-specific toxicity of the antisense oligonucleotides. This observation strongly suggests that the observed effects of the antisense oligonucleotides on embryogenesis are attributable to a specific knockdown of translation of the endogenous XCEP2 mRNA, and a concomitant reduction in XCEP2 protein.

In contrast to the morpholino knockdown effects, overexpression of XCEP2 in embryos by injection of up to 1 ng XCEP2 mRNA was found to result in no detectable abnormalities in embryonic development (data not shown). The absence of effects may not be unexpected, given current models of RhoGTPase effector protein activation. RhoGTPase effector proteins are activated by direct binding to GTP-bound RhoGTPases. As such, the number of activated RhoGTPase molecules may constrain the number of effector proteins bound and activated, regardless of the heightened expression levels of the effector protein.

### XCEP2 morpholinos do not interfere with mesoderm induction

**Figure 7:**
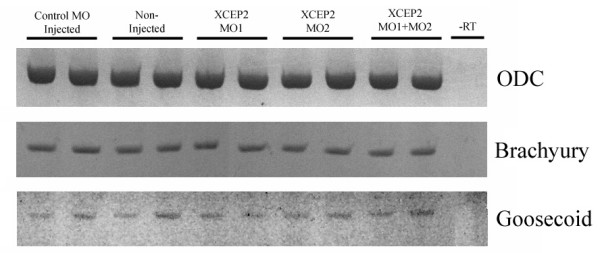
**Antisense XCEP2 morpholino oligonucleotides do not inhibit mesodermal induction. **Semiquantitative RT-PCR analysis was conducted on duplicate RNA samples using primers for Brachyury (a pan-mesodermal marker), goosecoid (a dorsal mesodermal marker) and ornithine decarboxylase ("ODC", used as loading control). For control and morpholino-injected conditions, 100 ng of morpholino oligonucleotide was injected into the animal region of 1-cell stage embryos.

The observed effects of antisense XCEP2 morpholinos in *Xenopus *embryos suggests that XCEP2 may play a direct role in directing the morphogenetic movements of gastrulation. This interpretation would be consistent with the limited characterization that have thus far been conducted on functional properties of this class of proteins [29,30]. However, it is also conceivable that XCEP2 antisense morpholinos may indirectly inhibit gastrulation by interfering with mesodermal induction. In order to assess this possibility, the level of expression of the pan-mesodermal marker, brachyury, and the dorsal mesodermal marker, goosecoid, was assessed in control and antisense morpholino injected embryos at gastrula stage using semi-quantitative RT-PCR (see Figure 7). Expression of mesodermal markers was similar in non-injected, control morpholino injected and antisense XCEP2 injected embryos, suggesting that it is the behavior of mesodermal cells during gastrulation (rather then the presence or absence of mesodermal cells) that is affected by XCEP2 antisense morpholino oligonucleotides.

**Figure 8:**
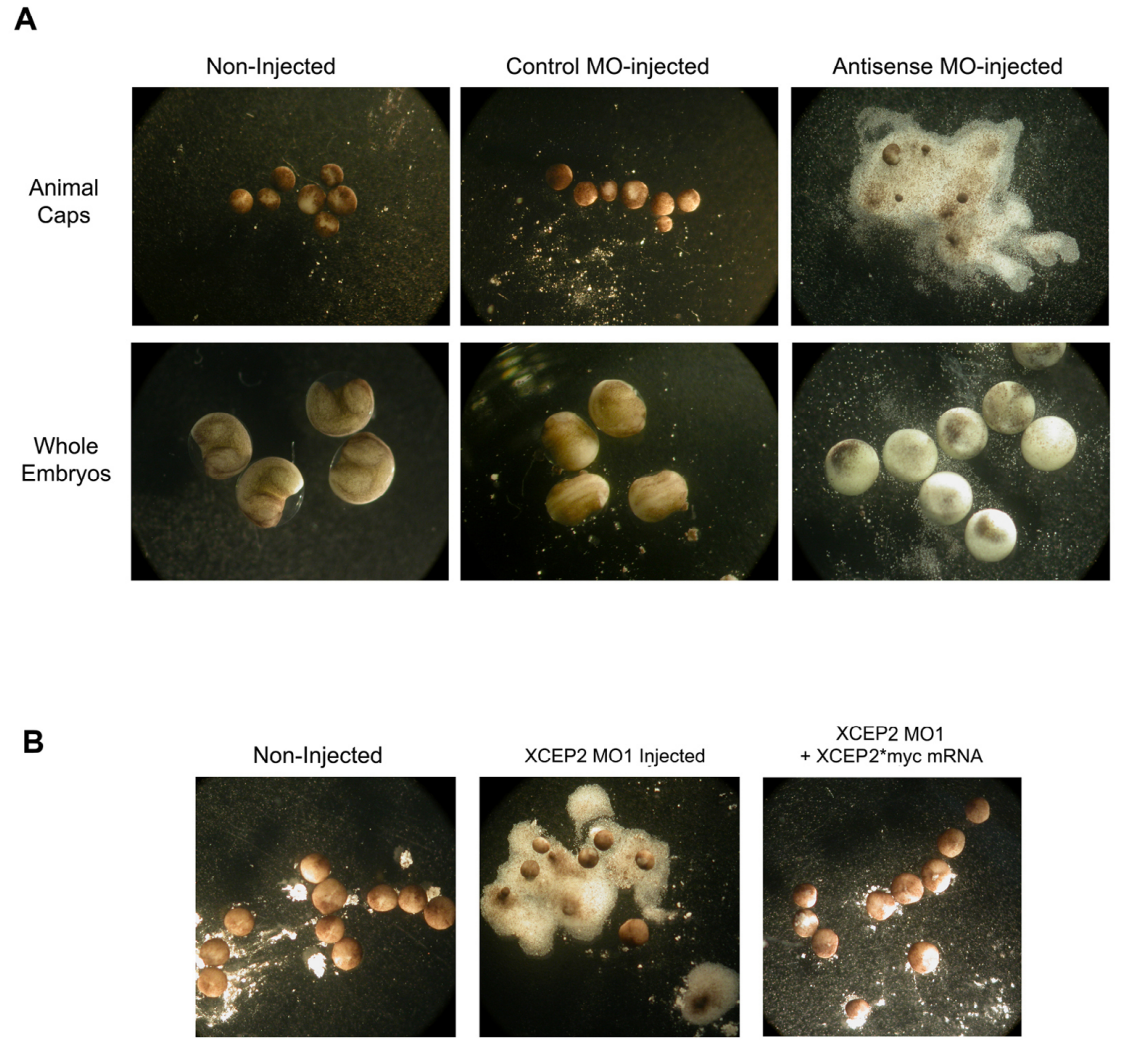
**Effects of antisense XCEP2 Morpholino oligonucleotides on animal cap explant cell adhesion. **(A) 100 ng morpholino oligonucleotide MO1 was injected into the animal region of each blastomere of 2-cell stage embryos. Animal cap explants from antisense MO1-injected embryos exhibited a marked loss of integrity (upper right panel) 24 hours after explantation, as compared to animal caps from non-injected or control morpholino injected embryos (upper left panels). Effects of these treatments on whole sibling embryos at Stage 22–23 are shown in the lower panels. In (B), embryos were injected with 75 ng of MO1 antisense morpholino in each blastomere at the 2-cell stage. Animal explants derived from non-injected embryos (left panel), MO1-injected embryos (middle panel), or embryos co-injected with 0.7 ng XCEP2*myc mRNA (right panel) are shown 24 hours after explantation.

### XCEP2 morpholinos Interfere with cell-cell adhesion in animal cap explants

In order to assess the mechanism by which XCEP2 influences gastrulation, the effects of XCEP2 morpholinos on cultured animal cap explants were evaluated. While the initial intent of these experiments was to discern whether XCEP2 morpholinos prevented normal activin-induced convergent extension of animal caps, it soon became clear that morpholino-treated explants had a more fundamental deficiency. Within a 24 hour period, animal cap explants derived from XCEP2 antisense morpholino (MO1) -injected embryos were found to completely lose their integrity (see Figure 8A, upper right panel), collapsing into piles of dissociated cells. This contrasts with control injected and non-injected caps, which remained tightly associated at 24 hours (see Figure 8A, upper left panels). This result was evident with or without activin treatment (data for activin treated caps is not shown). The observed defect in XCEP2 MO1 treated caps suggested a loss of cell-cell adhesion, as cells of disintegrated explants appeared to be completely dissociated, with few if any adherent clusters of cells. The observed loss of integrity followed a reproducible pattern. At approximately 18 hours after explantation, dissociated cells from the interior of the explant could often be observed discharging from the healed wound site (or, in some cases, at other localized sites), causing the explants to become progressively reduced in size. The cells of the outer pigmented epithelium of the explant remained adherent until the latest stages of the 24 hour time course, by which time they often also dissociated.

The animal cap dissociation caused by the MO1 morpholino could be largely rescued by co-injection with a XCEP2*myc mRNA (see Figure 8B), which has modifications that make it insensitive to translational inhibition by our morpholinos (see Figure 5B). This suggests that the dissociation observed is not a non-specific toxicity or cross-inhibition effect. XCEP2*myc mRNA rescued explants showed little difference from non-injected controls at and beyond the 24 hour time point.

## Discussion

The Rho GTPases and their associated effector proteins are known to play diverse roles in the regulation of cytoskeletal remodelling, cellular adhesion and cell motility. The complex morphogenetic movements associated with gastrulation in *Xenopus*, including changes in the morphology and polarity of mesodermal cells, and their later mesolateral intercalation of these cells during convergent extension, are now known to be dependent upon RhoGTPase functions [5,12,14] (for review, see [35,36]). Currently, however, there is little known regarding the identity or role of specific effector proteins that are utilized to convert changes in the GTP binding state of RhoGTPases into gastrulation-associated changes in cytoskeletal architecture, cell morphology, and cellular adhesion and migration.

The data presented here indicate a role for the *Xenopus *Cdc42 effector protein 2 (XCEP2) in gastrulation movements. XCEP2 is a member of the recently characterized CEP family of Rho GTPase effector proteins [29,30], which include the previously characterized mouse protein, MSE55 [33]. Effector proteins of the CEP family share a conserved expanded CRIB domain, which binds Rho GTPases, and two other highly conserved protein domains (CI and CII) [29,30]. By analogy to other Rho GTPase effector proteins, it has been proposed that Cdc42 binding to the CRIB domain of CEP proteins leads to a conformational change that exposes the previously inaccessible CI or CII domains (reviewed in [37]). The exposed domains of the effector protein would then be free to interact with downstream components of the signaling/regulatory pathway. When overexpressed in cultured cells, members of this family of effector protein induce marked pseudopodial/ lamellipodial extensions, membrane ruffling, alterations in actin and vinculin organization, and a reduction of E-cadherin staining at adherens junctions [29,30].

Consistent with a potential role for this class of proteins in embryonic morphogenesis, we have shown that XCEP2 expression is temporally regulated at gastrulation stages, when major modulations of cellular morphology, cytoskeletal organization, and cellular adhesion are occurring. mRNA for XCEP2 is present prior to mid-blastula transition, persists through mid gastrulation, and is strongly down-regulated by the time the blastopore closes and neurulation begins. This pattern would suggest that XCEP2 protein would be present through the period when active gastrulation movements are occurring. The diffuse spatial pattern of mRNA and, presumably, protein expression of XCEP2 may suggest that XCEP2 functions broadly in cells of the animal and equatorial regions. However, the observed broad spatial distribution of XCEP2 mRNA does not preclude the possibility that the XCEP2 protein may be functionally activated or inactivated at discreet times and locations during early embryonic development.

Furthermore, we show that antisense morpholino oligonucleotides capable of blocking translation of the XCEP2 message interfere with *Xenopus *gastrulation, delay the closure of the blastopore and inhibit embryonic elongation. The observed rescue with XCEP2 mRNA is strong evidence for the specificity of the antisense morpholino effect. These effects are not due to a loss of mesodermal induction, as brachyury and goosecoid expression do not change in response to antisense XCEP2 morpholinos. This is particularly relevant given recent reports demonstrating a direct link between brachyury expression and control of cellular migration [38].

The effects we report require relatively high, although not unprecedented, doses of morpholino antisense oligonucleotide. This dosage requirement may reflect the difficulty inherent in morpholino-mediated translational knockdown of maternally expressed genes. XCEP2 mRNA and (presumably) XCEP2 protein are present prior to midblastula transition. Given this situation, the timing morpholino induced protein downregulation is dependent both upon the effectiveness of translational blockade, and the half life of the protein in the cytoplasm. In this context, it may be essential to impose close to complete translational inhibition in order to reduce protein levels rapidly enough to affect early embryonic events, such as gastrulation.

Clearly, specific probes to assess endogenous XCEP2 protein expression will be necessary for fuller characterization of the role of this protein during gastrulation. For this reason, antibodies are currently being raised against the XCEP2 protein. These antibodies will be important in the characterization of the developmental time course of endogenous XCEP2 protein expression, assessment of the subcellular localization of the XCEP2 protein, isolation of potential XCEP2 binding partners, and in assessing and further optimizing the extent of protein down-regulation in morpholino injected embryos.

Currently, the specific mechanism by which XCEP2 exerts its role in gastrulation is unknown. However, our preliminary data suggest that XCEP2 may either contribute to a required "ground state" of cellular adhesion or play a role in modulations in the strength of cadherin-mediated cell-cell adhesion that are known to occur during gastrulation [22,39,40]. More detailed work will be necessary to clearly distinguish between these possibilities.

In embryos Wnt-mediated signals have been shown to activate Cdc42, a process that is required for normal gastrulation movements [7,12]. In future work, it will be important to discern whether XCEP2 plays an important role in transducing these upstream signals into changes in cellular behaviour during gastrulation.

The known functional properties of the CEP class of effector proteins, and the characteristics of CRIB domain effector proteins in general, suggest some interesting possibilities relating to the control of cell adhesion during gastrulation. Consistent with the observed functions of the XCEP2 homologs in cultured cells, XCEP2 in embryos may impinge on regulatory circuits downstream of Cdc42 that control actin filament assembly, which in turn may affect diverse cellular processes, including assembly of adherens junctions. Alternatively, XCEP2 may more directly impinge upon cadherin functional activity, perhaps by influencing the association of IQGAP or other molecules with cadherin complexes. In future studies, it will also be important to establish whether embryonic activation of whether there are links between Wnt-mediated activation of Cdc42 and functional activation of XCEP2 and to characterize the mechanism(s) by which XCEP2 contributes to cell adhesion between cells of gastrulating embryos.

## Conclusions

It has become clear in recent years that an integrated network of signals involving Rho GTPase proteins and their effector proteins help to control and regulate the diverse and intricate morphogenetic processes that occur during embryonic development. Less clear are the specific modes of functional interaction between the multiple Rho GTPases and the diversity of potential effector proteins. We have shown that XCEP2 is one component in the complex regulatory puzzle contributing to morphogenetic processes during *Xenopus *gastrulation. For this reason it will be interesting and important to discern further the role of XCEP2, with regard both to its relationship to intracellular signalling pathways and its effects on cellular behavior during gastrulation.

## Methods

### Restriction fragment differential display primer and adaptor sequences

Adaptor 1-

5'-ATGAGTCCTGAC-3' (upper strand)

5'-PO_4_-CGGTCAGGACTCAT-3'(lower strand).

Adaptor 2 (the 3' phosphate group of the lower strand inhibits DNA polymerase extension)-

5'-ACTGGTCTCGTAGACTGCGTACC-3'(upper strand)

5'-PO_4_-CGGGTACGCAGTC-PO_4_3' (lower strand).

"Universal" RFDD-PCR primer (complementary to Adaptor 2)-

5'-ACTGGTCTCGTAGACTGC-3'

"Selection" Primer 4 (complementary to Adaptor 1, with a 3-nucleotide 3' extension)

5'-ATGAGTCCTGACCGAAAG-3' (3-nucleotide extension is underlined)

### Primers used for construction of full-length XCEP2 cDNAs (sequences encoding start codon shown in bold, PCR-induced mutation sites are underlined)

XCEP2 cDNA forward-

5'-ATTGCAAAG**ATG**TCCGCCAAG-3'

XCEP2* cDNA forward-

5'-CGGGATCCTAG**ATG**TCGGCGAAAGCGCCGATATACCTAAAGAG AAG-3'

XCEP2 cDNA reverse-

5'-AACGTATCCCCTTCCCCA-3'

### RT-PCR primer sequences

XCEP2A forward (complementary to 5' untranslated region of the cDNA sequence)

5'-AACGTATCCCCTTCCCCA-3'

XCEP2A reverse (complementary to 5' untranslated region of the cDNA sequence)

5'-AAAGAGAAGTAGCCGTAAAGGA-3'

XCEP2B forward

5'-GCCAAGGCCCCGATATAC-3'

XCEP2B reverse

5'-CCAATAGCAGGTAGGGAA-3'

Brachyury forward

5'-GGATCGTTATCACCTCTG-3'

Brachyury reverse

5'-GTGTAGTCTGTAGCAGCA-3'

Goosecoid forward

5'-ACAACTGGAAGCACTGGA-3'

Goosecoid reverse

5'-TCTTATTCCAGAGGAACC-3'

ODC forward-

5'-GTCAATGATGGAGTGTATG-3'

ODC reverse

5'-TCCATTCCGCTCTCCTGA-3'

### Morpholino oligonucleotides

Antisense morpholino oligonucleotides, which specifically block translation of targeted mRNAs [41] were synthesized by GeneTools, LLC (Philomath, OR) and were designed to interact with both characterized pseudoalleles of the XCEP2 gene. Antisense oligos were targeted to the region upstream and downstream of the translational start site of the XCEP2 mRNA. The target sequences that were chosen were compared to the Genbank database to confirm that the XCEP2 antisense oligonucleotides would not be expected to interfere with the expression of other known gene products. The morpholino sequences utilized in these studies were as follows, with morpholino sequence complementary to the start codon of the cognate mRNA shown in bold:

Antisense XCEP2 MO1 (specific to the XCEP2A, characterized in this study)-

5'-GGGCCTTGGCGGA**CAT**CTTTGCA-3'

Antisense XCEP2 MO2 (specific to XCEP2B, *Xenopus *Genbank sequence accession # BC045241)-

5'-GGGCCTTGGCTGA**CAT**CTTTCCA-3'

Control Morpholino oligonucleotide

5'-CCTCTTACCTCAGTTACAATTTATA-3'

### Restriction fragment differential display PCR, gene identification and isolation

Restriction Fragment Differential Display PCR (RFDD-PCR) procedures were patterned closely after the commercially available DisplayProfile kit from QBiogene (Carlsbad, CA), with minor modifications. In this differential display procedure, Adaptors 1 and 2 (see **RFDD-PCR primer and adaptor sequences**, above) are ligated to restriction digested, double stranded cDNA. Amplification of this cDNA is conducted with the "universal primer", complementary to one of the adaptor sequences, and one of 64 "selection" primers that bind largely to the to the second adaptor sequence but have a 3-nucleotide 3' extension that is complementary only to a subset of cDNA inserts. An extension-inhibiting chemical modification of the lower strand Adaptor 1, consisting of a 3' phosphate group in our modified procedure, and the 3-nucleotide extension of the selection primer largely restricts amplification to cDNA fragments that have different adaptor sequences ligated on opposite ends. Furthermore, any particular selection primer will selectively amplify only a subset of cDNA fragments (in theory, approximately 1/64^th ^of the total).

Total RNA from staged, duplicate batches of *Xenopus laevis *embryos was purified using Trizol reagent (Invitrogen, Carlsbad, CA). RNA from *Xenopus laevis *embryonic stages 7, 8.5, 9.5, 10.5 and 12.5 were prepared. First strand cDNA was synthesized using oligo dT primer and Superscript III reverse transcriptase (Invitrogen), followed by second strand synthesis catalyzed by DNA polymerase I (Roche, Indianapolis, IN). Double stranded cDNA was purified using GeneClean silica resin (Qbiogene, Carlsbad, CA), and then digested with TaqI restriction enzyme (Roche, Indianapolis, IN). An annealed mix of RFDD-PCR of Adaptor 1/Adaptor 2 oligonucleotides was added, and ligated to the TaqI digested cDNA ends using T4 DNA Ligase (Roche). Touchdown PCR using the universal RFDD-PCR primer, ^35^S-labelled dCTP, and "Selection" Primer 4 was conducted using Accuprime PCR mix (Invitrogen), with the following cycling parameters:

Pre-dwell: 94°C 4 minutes

10 cycle touchdown PCR, with a 0.5 temperature decrement each cycle:

94°C 30 seconds

60°C→ 55°C 30 seconds

72°C 1 minute

30 cycle standard amplification:

94°C 30 seconds

55°C 30 seconds

72°C 30 seconds

Post dwell: 72°C, 5 minutes

Samples were run on a standard 6% denaturing DNA sequencing gel, and the gel was dried and subjected to autoradiography. Bands showing differential expression were excised from the gel and eluted by heating at 95°C for 15 minutes. Eluted fragments were re-amplified with 30 cycles of standard PCR, using the original primers (above). Direct cycle sequencing of isolated product (Thermosequenase; United States Biochemical, Cleveland, OH) revealed that one of the fragments corresponded to the 5' end (overlapping the translational start site) of a *Xenopus *homologue of the Cdc42 Effector Protein 2, which we refer to as "XCEP2". The full length cDNA was amplified using gene-specific primers and poly-dT primers using standard 3' RACE procedures [42]. The sequence of the full-length cDNA was determined by commercial automated sequencing (MWG Biotech, High Point, NC).

### Plasmid construction and RNA preparation

The RACE amplified full-length fragment isolated from the RFDD-PCR procedure (see above) was cloned into the pCR2.1 vector, using the pCR2.1 TA cloning kit (Invitrogen), and commercially sequenced (MWG Biotech). To construct a vector encoding XCEP2A fused to a C-terminal 6xmyc tag, PCR was conducted using primers directed to the 5' and 3' end of the XCEP2A cDNA sequence (XCEP2A cDNA forward and XCEP2A cDNA reverse) with the cloned, full length XCEP2A as template. The primers used in this PCR mutated the normal stop codon and introduced a BamHI and ClaI site at the 5' and 3' ends of the amplified product, respectively. BamHI/ClaI digested full length cDNA was directionally cloned in the sense orientation into the corresponding into BamHI/ClaI digested pCS2-myc vector, producing the plasmid pCS2-XCEP-myc. In this context, the full length X-CEP2A cDNA encodes a fusion protein with a C-terminal 6x myc tag. A second construct, containing introduced mutations in the XCEP2A antisense morpholino target region, was also derived by PCR. To construct this vector, PCR was conducted using XCEP2* forward and XCEP2 reverse primers with the full-length XCEP2 DNA as template. XCEP2* forward introduced multiple changes in the sequence of the cDNA encoding the translational start site region (see Figure 5A). These changes do not change the predicted amino acid sequence of the encoded protein, but would be expected to significantly reduce or eliminate binding of the antisense morpholino oligonucleotides used in this study.

For transcription of antisense probe for *in situ *hybridization, the full length X-CEP2A cDNA was subcloned into the pCS2+ plasmid in an antisense orientation (relative to the SP6 promoter), producing the plasmid pCS2-XCEP2-anti. In a similar fashion, an RNA expression vector containing the XCEP2 insert in the sense orientation in pCS2+ was also constructed.

Capped mRNAs were synthesized *in vitro *using the mMessage mMachine kit (SP6) from Ambion (Austin, TX), and were dissolved in distilled water prior to injection.

### Semi-quantitative RT-PCR

Total RNA was isolated from embryos using Trizol (Invitrogen) according to manufacturer's instructions. Reverse transcription was carried out using SuperscriptIII (Invitrogen). Assessment of relative levels of gene expression over developmental time, or under different experimental conditions, was carried out using standard semi-quantitative RT-PCR methods [43], with the primers previously described (see **RT-PCR Primers**, above). 25 cycles of PCR were carried out using Accuprime PCR mix (Invitrogen) in 25 ul reactions containing 2 ul of 200 ug/ml reverse transcribed cDNA, 0.3μM of each primer, and a trace of radiolabelled nucleotide to allow for autoradiographic visualization. Parallel reactions containing primers specific to the metabolic gene, ornithine decarboxylase (ODC), were used as a loading control in all semi-quantitative RT-PCR experiments. RT-PCR products were separated electrophoretically on 5% acrylamide gels, which were dried and subsequently subjected to autoradiography. RT-PCR of the XCEP2A allele resulted in stronger amplification than with the XCEP2B primer set. XCEP2B bands required correspondingly longer exposure times (2–4 fold) for detection.

### Embryo manipulations

Adult *Xenopus laevis *were purchased from Nasco (Fort Atkinson, WI). Eggs were obtained and fertilized according to standard methods. Staging was determined according to Nieuwkoop and Faber [44]. Antisense morpholino oligonucleotides and *in vitro *transcribed mRNA's were injected into two-cell stage embryos, with injected volumes of approximately 13 nl in doses as described. All injections were carried out using the Nanoject positive displacement microinjector (Drummond Scientific, Broomall, PA).

Measurement of blastopore to embryo diameter ratios was accomplished by dividing the measured blastopore diameter (the distance between dorsal and ventral poles of the blastopore) by the embryo diameter. Diameter measurements were obtained using digital images of individual embryos, and the "Ruler" tool of the Adobe Photoshop software.

To assess the effects of morpholino antisense oligonucleotides on animal cap explants, morpholino injected and control embryos were allowed to develop until stage 8.5. At stage 8.5 animal caps were dissected, and then cultured in 1xMMR for 24 hours. Non-dissected sibling embryos were allowed to develop until post-neurula stages in order to asses the effectiveness of the morpholino treatment.

### Detection of myc-tagged protein expression

Proteins from embryos were extracted in 10 volumes of isotonic buffered saline containing 1% NP40. Extracts were cleared by a brief centrifugation, and supernatants were denatured by a 5-minute incubation at 95°C after diluting samples with SDS-PAGE sample buffer. Proteins were separated on 8–16% gradient polyacrylamide gels, and transferred to nitrocellulose. After blocking, blots were probed with monoclonal antibody 9E10 [45], which recognizes the myc epitope, and AP-conjugated goat-anti-mouse IgG (Biorad, Hercules, CA). Antibody binding was detected using Enhanced Chemiluminescence (ECL) reagents (Amersham-Pharmacia, Piscataway, NJ). Equivalency of protein loads was confirmed by running equivalent volumes of protein extracts on SDS-PAGE, and staining overnight with SYPRO Ruby (BioRad), with detection and documentation of protein bands conducted using UV transillumination.

### Wholemount *in situ *hybridization

Digoxygenin-labelled antisense RNA probe was transcribed *in vitro *from linearized pCS2-XCEP2anti plasmid using a digoxygenin nucleotide labeling mix (Roche) and the mMessage mMachine *in vitro *transcription kit (SP6) from Ambion. *In situ *hybridization was carried out essentially as described [46], using non-hydrolyzed X-CEP2 digoxygenin-labeled probe. Embryos were subjected to a hybridization temperature of 62°C. AP-conjugated anti-digoxygenin FAb fragments (Roche) and BM Purple substrate (Roche) were used to detect hybridized probe.

## Authors' contributions

KKN played a critical role in establishing *in situ *hybridization methodology in the laboratory, and conducted *in situ *hybridizations. RWN conceived of the study, designed and conducted molecular biological, embryological and biochemical experiments, and wrote the manuscript. Both authors read and approved of the final manuscript.
